# Common barriers and facilitators to the implementation of digital medicine in chronic respiratory diseases: a systematic review of reviews

**DOI:** 10.3389/fdgth.2026.1927835

**Published:** 2026-10-26

**Authors:** Julia Korel, Sebastian Kuhn, Rainer Gloeckl, Rembert Koczulla, Michael Dreher, Beate S. Müller, Ragnar Hirschbrich, Erwin Junker, Tobias Hastenteufel, Xavier Llauradó, Zouhair Haddi, Claus F. Vogelmeier, Gernot Rohde, Nurlan Dauletbaev

**Affiliations:** 1 Department of Internal, Respiratory and Critical Care Medicine, University Hospital Giessen and Marburg, German Center for Lung Research (DZL), Philipps-University of Marburg, Marburg, Germany; 2 Institute for Digital Medicine, University Hospital Giessen and Marburg, German Center for Lung Research (DZL), Philipps-University of Marburg, Marburg, Germany; 3 Institute for Pulmonary Rehabilitation Research, Schön Klinik Berchtesgadener Land, Schönau am Königssee, Germany; 4 Department of Pulmonary Rehabilitation, German Center for Lung Research (DZL), Philipps-University of Marburg, Marburg, Germany; 5 Teaching Hospital of the Paracelsus Medical University Salzburg, Salzburg, Austria; 6 Medizinische Klinik V, Klinik für Pneumologie und Internistische Intensivmedizin, Uniklinik RWTH Aachen, Aachen, Germany; 7 Institute of General Practice, Faculty of Medicine and University Hospital Cologne, University of Cologne, Cologne, Germany; 8 Qurasoft GmbH, Koblenz on the Rhine, Germany; 9 NVISION Systems and Technologies, Barcelona, Spain; 10 Department of Pediatrics, Faculty of Medicine, McGill University, Montreal, QC, Canada; 11 The Research Institute of McGill University Health Centre, Montreal, QC, Canada; 12 Faculty of Medicine and Healthcare, Al-Farabi Kazakh National University, Almaty, Kazakhstan

**Keywords:** barriers, bronchial asthma, chronic obstructive pulmonary disease, co-occurrence network analysis, cystic fibrosis, facilitators, respiratory medicine, systematic review of reviews

## Abstract

**Background and research question:**

Digital Medicine offers significant potential for improving care in Respiratory Medicine, yet its full uptake remains limited. Barriers and facilitators involve multiple stakeholders and are likely shared across diseases and technologies. This study had the following research question: What are the common barriers, facilitators, and transferable concepts for implementing Digital Medicine in respiratory diseases, exemplified by bronchial asthma, cystic fibrosis (CF), chronic obstructive pulmonary disease (COPD), and COVID-19?

**Study design and methods:**

Systematic review of reviews. Three electronic databases (PubMed, Scopus, and Web of Science), as well as Cochrane Library were searched for review publications from September 2019 through May 2026. Nine included reviews were appraised using AMSTAR-2. Original publications referenced within these reviews were additionally screened for suitability for data extraction, leading to inclusion of 18 original publications. Extracted barriers and facilitators were visualized as disease- and actor-stratified co-occurrence networks and mapped to two implementation frameworks (CFIR and Chaudoir et al. 2013).

**Results:**

No COVID-19 publication met inclusion criteria. Asthma and, partially, CF evidence was rich and included institution-level data, while COPD evidence was less diverse. One CF program survey had structured its data according to the CFIR framework, enabling uniquely complete mapping of barriers and facilitators.

**Conclusion:**

High-quality, framework-structured, network-visualizable evidence on barriers and facilitators to Digital Medicine exists in bronchial asthma and, partially, CF. In contrast, evidence on barriers and facilitators in COPD is not yet systematic. Future studies should be anchored in frameworks for sufficient comprehensiveness and facilitated knowledge translation into implementability.

**Systematic Review Registration:**

https://www.crd.york.ac.uk/PROSPERO/view/CRD420251070944, PROSPERO, CRD420251070944.

## 1 Introduction

Digital Medicine is a fast-paced field of clinical medicine that employs diverse digital approaches in order to promote health and alleviate disease. Digital Medicine promises more efficient and, on a longer run, better individualized clinical approaches. Importantly, Digital Medicine is also an enabler of patient autonomy, since many diagnostic procedures and interventions are now feasible directly in patients' homes.

Increased utilization of Digital Medicine is universally considered as beneficial, yet its full potential is far from being reached. Effective and sustained uptake of Digital Medicine-based clinical approaches requires substantial restructuring of established clinical workflows. It is important to recognize that Digital Medicine implicitly promotes the patient from a mere recipient to a key stakeholder and important actor. This transformation has direct implications for Respiratory Medicine, the medical specialty that often caters to the extremes of the human age span. These are the age groups that are characterized by the highest disease vulnerability and a clear need for the home-centered approaches, such as remote monitoring. At the same time, the most vulnerable population subgroups are also the ones with a troublesome deployability of the digitally anchored clinical approaches.

Besides patients and their families and healthcare professionals, the lineup of key stakeholders includes technology developers, experts in data safety, data privacy, and digital ethics, as well as policymakers. This extended stakeholder lineup justifies a comprehensive and holistic approach to uptake of Digital Medicine. In practical terms, this means that increased understanding of the factors that define the uptake, either positively (as “facilitators”) or negatively (as “barriers”), requires a consideration of all key stakeholders.

Notably, extensive experience with deployment of Digital Medicine, either empirically or informed by implementation frameworks, already exists across respiratory diseases and conditions since the COVID-19 pandemic, when Digital Medicine enjoyed a universal and strong support. While each respiratory disease has its own particularities, it is plausible that many of the barriers and facilitators are shared across diseases. Subsequently, identification and characterization of potential disease-agnostic facilitators and barriers would be highly beneficial, especially, if the aforementioned key stakeholders are considered as allied actors.

Since implementation of Digital Medicine requires a concerted effort of key stakeholders, mapping their respective and mutual barriers and facilitators requires a validated structure, like an implementation framework. Such mapping, while increasing our theoretical understanding of complex, multi-stakeholder defined processes, would also have direct practical value. Policymakers could use it to focus on specific challenges that are shared across diseases, or across stakeholders, or within each stakeholder. Furthermore, joint implementation efforts of patients and their families, healthcare professionals, and technology developers, and other stakeholders could be concentrated on overcoming few critical and highly connected sources of implementation delays. Moreover, a systematic mapping of knowledge gaps would inform future research to expand both the theory and practical aspects.

It is typical for research in barrier and facilitator discovery to have the outputs in a tabular format. Since the aforementioned factors may involve more than one stakeholder, especially, in a complex manner (e.g., as an interdependency), such tabular representation is insufficient for a discovery of the underlying interconnectivity. A better representation is feasible if barriers and facilitators are presented as a network. Such network representation can map the barriers and facilitators as points called “nodes”. The joint appearance of certain nodes with other barriers or facilitators – or across stakeholders – can be defined as a co-occurrence, indicated by a line. In this manner, a network analysis can expose an interconnectivity that is not immediately evident in a tabular format.

These considerations motivated the present umbrella review (systematic review of reviews). The research question was defined as follows: What are the common barriers, facilitators, and transferable concepts for implementing Digital Medicine in respiratory diseases, exemplified by bronchial asthma, cystic fibrosis (CF), chronic obstructive pulmonary disease (COPD), and COVID-19? To visualize the commonalities, this review employed two parallel approaches: (i) via building a co-occurrence network representation (done separately for each targeted disease) of published barriers and facilitators, as well as other contributors, and (ii) mapping the barriers, facilitators, and other contributors to domains of validated implementation frameworks (1, 2). To ensure factual and contextual fidelity of barrier/facilitator/other contributor identification, this review screened and evaluated both the identified review publications and the original referenced within those reviews.

The findings of this umbrella review, including mapping of identified barriers and facilitators to established frameworks, and demonstration of areas of current evidence sparsity, are presented in the subsequent text.

## 2 Study design and methods

### 2.1 Reporting standards

This umbrella review followed the PRISMA 2020 statement (3) and the Synthesis Without Meta-analysis (SWiM) guideline. The review was registered in PROSPERO before the process of publication identification and screening began (CRD420251070944).

### 2.2 Search for review publications and eligibility criteria

PubMed, Scopus, and Web of Science were primary sources to identify relevant review publications. Examples of PubMed search strategies are presented in Supplementary Table S1A–S1D. The Cochrane Library was searched for potential publications not identified by the aforementioned three sources. This was supplemented by reference-list checking of included reviews, as well as a search of trial and study registers. For initial inclusion, published reviews had to be in English language and with publication dates between 1 September 2019 and 31 May 2026. Identified reviews were downloaded, deduplicated in Endnote [v20 (4); “Bramer deduplication” (5)], and screened (title-abstract screening) in Rayyan (6) against the following criteria: language, studied population (bronchial asthma, CF, COPD, or COVID-19), and the area of Digital Medicine utilization. Digital Medicine was defined broadly and encompassed the concepts of remote patient monitoring, electronic patient-reported outcome collection, and mobile-health videoconsultation (telehealth). A pre-defined keyword set [“telemedicine,” “remote patient monitoring,” “mHealth,” “wearables,” “ePRO(M),” “PRO(M),” “videoconsult*”] was drawn from the authors' previous similar projects in this area (7). At the title-abstract screening stage, a review was to be excluded only when it contained no mentioning of “barrier”, “facilitator”, or “transferable concept” in association with the aforementioned Digital Medicine concepts. Identified potentially relevant reviews of diverse types (systematic review, meta-analysis, scoping review, expert panel summary, or narrative review) proceeded to the next stage of selection. This screening strategy deliberately emphasized recall over specificity in order to ensure adequate coverage. Review publications that passed title-abstract screening were evaluated as full texts against pre-defined and more stringent selection criteria. In particular, to be included, a review had to offer a substantive, clearly attributable text subsection that is readable by a human and machine-searchable (e.g., via keyword search of a full-text PDF) and had descriptions of barriers/facilitators/transferrable concepts. Moreover, inclusion criteria also required that original publications that were referenced by a particular review for descriptions of barriers and facilitators presented a clear stakeholder-attributable source for the barriers and facilitators. This process is described in detail in subsequent subsection. Each included review was appraised using AMSTAR-2 (8).

Outcomes that were deemed as “transferrable” factors or concepts were those with relevance across at least two of the included diseases. In particular, such factor or concept should generalize beyond disease-specific pathophysiology or care pathway: e.g., digital literacy or family/caregiver support as opposed to fatigue that is a characteristic for Long COVID.

### 2.3 Two-step review appraisal process and rationale

Review appraisal proceeded in two subsequent steps: (i) appraisal of actual identified review publication and (ii) appraisal and extraction of evidence from original publications referenced within the review publication in question. The rationale for this two-step appraisal procedure was the following. First, this procedure alleviated a specific risk of review-level synthesis. This synthesis routinely pools findings from several primary studies into single summary sentences. During aggregation, the original context of a finding may inadvertently be lost. For example, a review publication may aggregate reports of actual experiences of barriers and facilitators with reports of perceptions of the aforementioned factors, or even expert opinions of such perceptions. Second, evidence aggregation at the level of a review publication may conflate the stakeholders. A particular example is when the reported patient-centered barriers and facilitators to Digital Medicine are actually collections of healthcare professionals' perceptions of such barriers and facilitators. Extracting the evidence directly from original publications' references by an appraised review publications avoids this context loss. Following initial testing, the appraisal of included review publications was limited to descriptive synthesis and utilization of the AMSTAR-2 scale. The mapping of stakeholder-stratified barriers and facilitators was based exclusively on the evidence extracted directly from appraised original publications.

This stringent two-step appraisal meant that a review publication could be included in this umbrella review only if at least one original publication, referenced by this review, also satisfied specific inclusion criteria:

- First, original publication had to report at least one relevant barrier, facilitator, or transferable concept within its Results section. All mentioning of barriers, facilitators, and transferable concepts outside of the Results section was ignored. This measure aimed to preclude accidental evidence from collateral sources or speculations.
- Second, a stakeholder source (e.g., a patient or research nurse) had to be clearly identifiable.
- Third, reporting of barriers and facilitators had to generalize beyond simple acceptability testing of a device, technology, or platform. In practical terms, the authors looked for evidence richer than System Usability Scale (9) or similar Likert-based scales. This requirement of deeper research aimed to identify the underlying factors and potential interconnectivity. *Example 1*, within a stakeholder: a belief of insufficient digital literacy (patient's subjective barrier) aggravates their actual low “digital literacy” (patient's objective barrier); this is compounded by “data privacy concerns” (patient's subjective barrier) and “technology-related challenges” (patient's objective barrier); this can be resolved by “patient education” (objective facilitator) and “prior experience with digital technology” (objective facilitator). *Example 2*, across key stakeholders: patient reports that “questions not answered by care team” is an objective barrier on patient side (connection to a healthcare professional); this can be compounded even further by “equipment failure” (technological barrier) and “financial adversity” (circling back to patient's objective barrier). Entangling such interconnectivity required granular data stratification, which was only feasible by original publication appraisal.

The scope of original publications was limited to the past ten years, which was a pragmatic feasibility decision. Following the screening of original publications and final inclusion, both the review publications and referenced original publications were subjected to data extraction. On a review side, this involved collection of review type and PMID, first author's family name, year of publication and journal, disease(s) covered, and the main domain of Digital Medicine addressed [e.g., mobile (m)Health or telemedicine]. For the purpose of this umbrella review, definitions of eHealth (delivery via a PC/website), mHealth (delivery via a mobile device), and telemedicine (telerehabilitation: delivery via video, typically in a synchronous mode) were similar to those published previously (10). Potential further technologies that may have been employed in original publications were Internet of Things (IoT) devices that independently collect, store and send patient data wirelessly (11) and Artificial Intelligence [AI, typically represented by a general purpose generative or Large Language Model (12)]-enabled chatbots. Additionally, collected review-level data included a number of original publications appraised by a review, as well as a short narrative description of reported barriers, facilitators, and transferable concepts.

Following this, original publications referenced within the included review publications were subjected to data extraction and aggregation. Specifically, each original publication was screened for the presence, veracity, and stakeholder-attributability of the reported barriers and facilitators, transferrable concepts and recommendations. The data were extracted separately for patients (including families and caregivers) and healthcare professionals across pre-defined categories. The latter included subjective and objective barriers and facilitators, incentivization, technological barriers and facilitators, as well as equity dimensions (as patient-centered factors). Institutional barriers and facilitators were compiled separately. Unclassifiable barriers and facilitations, as well as Technology Acceptance Model, a popular framework in this field (13) [modification: (14)], were extracted separately.

Each of these aforementioned fields was populated with a keyword or short phrase (tokenization) that corresponded to either (i) direct definition as stated in the original publication or (ii) universally accepted terminology (e.g., “digital literacy” or “digital infrastructure”). The aforementioned equity-related findings were categorized using PROGRESS-Plus (https://methods.cochrane.org/equity/projects/evidence-equity/progress-plus). The extracted evidence was assigned to one or more of its categories: (i) place of residence; (ii) race, ethnicity, culture, or language, (iii) occupation; (iv) gender or sex; (v) religion; (vi) education; (vii) socioeconomic status; (viii) social capital; or (ix) “Plus” (personal characteristics not covered by other categories). Each original publication was further classified by design, with a possibility to assign more than one tag, including technology testing, observational, randomized controlled trial, survey, or qualitative/mixed-methods study.

### 2.4 Framework mapping: consolidated framework for implementation research (CFIR)

In addition to horizontal network representation of the interconnectivity of barriers and facilitators, a hierarchical mapping was expected to yield informative outputs. Hierarchical mapping can be done within structures of implementation frameworks (typically: domains and constructs). The investigators' initial intention was to map identified evidence onto the constructs and domains of the Consolidated Framework for Implementation Research (CFIR) implementation framework (2). This framework was selected after comparison against four alternatives, each found unsuitable for a distinct reason. In particular, Normalization Process Theory (https://normalization-process-theory.northumbria.ac.uk) describes a process rather than classifying determinants; RE-AIM (https://re-aim.org/) classifies outcomes rather than barriers; the COM-B (Capability, Opportunity, Motivation–Behaviour) model (15) has no institutional or technological category; and the (integrated) i-PARiHS (Promoting Action on Research Implementation in Health Services) framework (16) is less deterministic than CFIR.

Of high importance to conclusions of this umbrella review, one identified original publication (described in detail in Results section) provided evidence on the CF-centered barriers and facilitators to Digital Medicine. Following identification of this publication, the investigators extracted relevant information and recapitulated the bar graph as a range plot to ensure correctness of data extraction. This was followed by a hierarchical network (dendronetwork) mapping that is presented and described in Results section.

### 2.5 Framework mapping: Chaudoir domains

Additionally utilized framework was an alternative framework posited by Chaudoir et al. (1). This framework has a five-domain structure that is arranged as (i) innovation/technology (e.g., mHealth or telemedicine), (ii) patient, (iii) provider (i.e., healthcare professional), (iv) organizational, and (v) structural. The investigators especially valued the fact that Chaudoir et al. framework's domains included a patient (unlike CFIR); this was specifically fitting for the data accumulated by this umbrella review.

### 2.6 Network construction

Barriers and facilitators were represented as co-occurrence networks, built separately for patients and for healthcare professionals using the data extracted from original publications. Each identified barrier or facilitator was drawn as a point (i.e., a node). The nodes were connected when they were reported together within the same publication.

Networks were analyzed by two criteria: (i) identification of the nodes carrying the most connections, which indicated the most consistently reported barriers and facilitators, and (ii) identification of the nodes that bridge separate clusters. Overall, this analysis aimed to identify the nodes that, if targeted, could plausibly affect more than one area of difficulty at once. Networks were built separately for each disease and stakeholder group.

### 2.7 Use of computational tools and Artificial Intelligence

All analyses were carried out in R [v. 4.5.0 (17)], using R Studio 2026.01.1 (18). The analyses utilized both base R functions and specialized packages (19–23).

Final stages of data analysis (that is, following compilation of the dataset extracted from original publications) were performed by the investigators with support of an Artificial Intelligence (AI) assistant (Claude 4.6 Sonnet; Anthropic, 2026). The latter was used for spot cross-checking of extraction fidelity against a sample of the investigators' own extractions and for identifying specific discrepancies in investigators' review. Claude-AI assistant was also used in the iterative development of the network visualization approach and its R implementation, including testing of alternatives the investigators had developed independently and in parallel to discussions with Claude-AI assistant. All images presented in this study are human-generated and are created using the R-based analytical and visualization pipelines. Claude-AI generation of images was never requested. All Claude-AI generated texts and information were thoroughly analyzed, independently verified, and rewritten by the investigators.

## 3 Results

### 3.1 Screening of review publications

The search (PRISMA flowchart: Figure 1) identified 278 potentially relevant review publications. Substantial proportion of review publications addressed more than one disease without clearly attributing individual findings to a single disease (77 publications), or addressed a Digital Medicine technology with a disease named only as background context. Among the remainder, COVID-19 and Long-COVID and COPD were the most frequently named respiratory diseases (respectively, 111 and 94 publications), followed by bronchial asthma (63 publications), and CF (10 publications). Additional publications addressed both asthma and COPD, or asthma and COPD together with a third condition, or asthma as a general example of a chronic diseases jointly with non-respiratory diseases. One publication concerned allergy.

**Figure 1:**
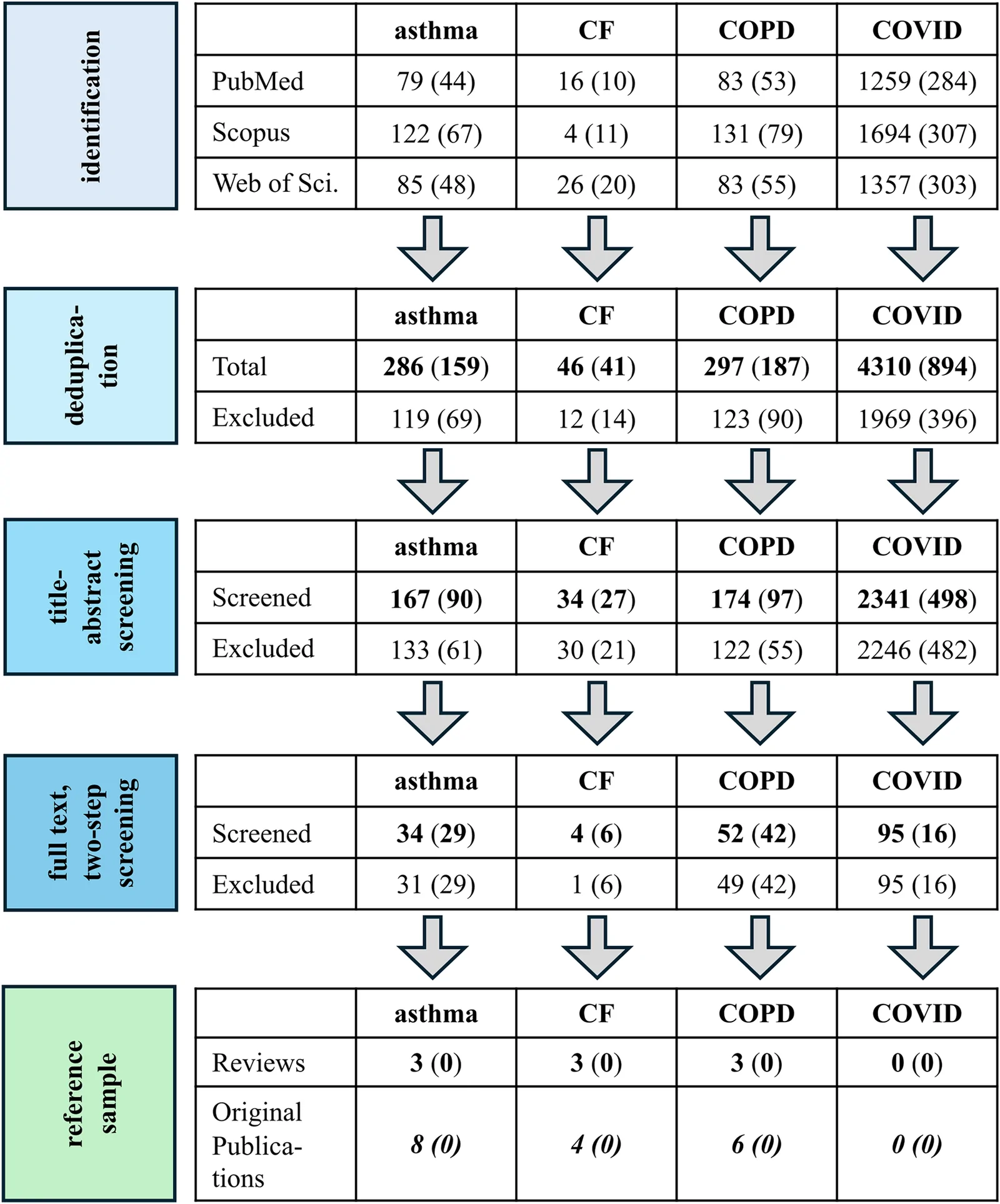
PRISMA flowchart. Numbers without parentheses reflect the number of references identified during initial screening (2020/01/01–2024/12/31). Numbers within parentheses reflect the number of references identified in additional literature search (2025/01/01–2026/05/31).

Of the 278 appraised review publications, 9 (Table 1) successfully passed the two-step appraisal procedure (i.e., as review publication and on the basis of referenced original publications). Four were systematic reviews, 2 were expert opinion or narrative reviews, 2 were scoping reviews, and 1 was a meta-analysis. The included reviews focused on the three diseases: asthma, COPD, and CF. Interestingly, no review that focused on COVID-19 was able to pass through the screening. The timeframe of included reviews spans between 2022 and 2024. The AMSTAR-2 appraisal summary is presented in Figure 2.

**Table 1:** Characteristics of included review and original publications.

No.	First author	Journal	Year	Disease area	Review type	PMID and citation	PMID of included original publications and citation
1	Uzzaman	Journal of Medical Internet Research	2024	Asthma	Meta-analysis	39626243 (36)	31181221 (30), 27357835 (31), 34309568 (32)
2	Hakizi-mana	Expert Review of Respiratory Medicine	2024	Asthma	Expert opinion/narrative review	38992946 (37)	36550136 (33), 36603013 (38)
3	Chike-Harris	Telemedicine Journal and e-Health	2024	Asthma	Systema-tic review	37624652 (39)	33940203 (27), 34152858 (25), 33730979 (26)
4	Vagg	Journal of Cystic Fibrosis	2023	CF	Systema-tic review	37230808 (40)	33106755 (34)
5	Peckham	Seminars in Respiratory and Critical Care Medicine	2023	CF	Expert opinion/narrative review	36535666 (41)	34930537 (28), 34930543 (42)
6	Yap	Internet Journal of Allied Health Sciences and Practice	2022	CF	Systema-tic review	Yap2022 (43)	31694805 (44)
7	O’Connor	Journal of Advanced Nursing	2025	COPD	Systema-tic review	39420778 (45)	33034565 (46)
8	Rama-chandran	Heart and Lung	2023	COPD	Scoping review	36801546 (47)	30154650 (48), 33262925 (49)
9	Wilde	Journal of Chronic Obstructive Pulmonary Disease	2022	COPD	Scoping review	35132933 (50)	32490838 (35), 30578215 (51), 29903700 (52)

CF, cystic fibrosis; COPD, chronic obstructive pulmonary disease. Publications without PMID are identified by first author's family name and year.

**Figure 2:**
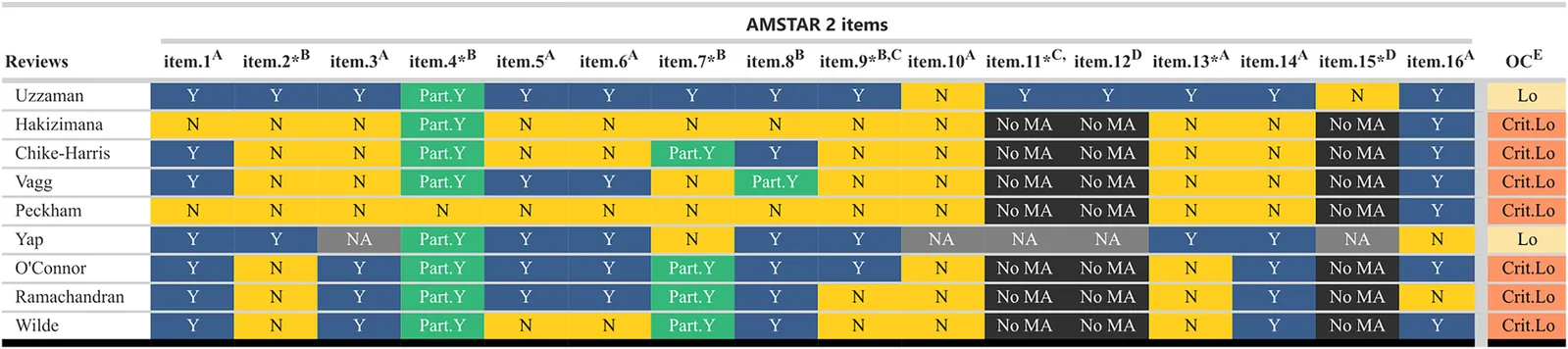
AMSTAR-2 appraisal summary. **(A)** AMSTAR-2 (8). PICO, participant, intervention, comparison, outcome; RoB, Risk of bias. *Asterisk indicates a critical item (domain). **(A)** Possible responses: Yes [Y]/No [N]. **(B)** Possible responses: Yes/Partial Yes [Part. Y]/No. A “Partial Yes” response is evaluated positively to the overall confidence rating. **(C)** Item response depends on separate assessment of randomized controlled trials (RCTs) and non-randomized studies of healthcare interventions (NRSI) in the review. **(D)** Possible responses: Yes/No/No MA. A “No MA” response stands for “no meta-analysis conducted” and does not affect the overall confidence rating. **(E)** Critically Low [Crit.Lo]/Low [Lo]/Moderate/High confidence in the results of the review. Critically Low: more than one critical flaw with or without non-critical weaknesses, Low: One critical flaw with or without non-critical weaknesses, Moderate: No critical flaws but more than one non-critical weakness, High: No flaws at all or one non-critical weakness.

Umbrella reviews often suffer from citation overlap, such that several reviews in the reference sample cite the same original publications. The estimate of general citation overlap, applied before selection of referenced original publications to 9 included reviews, was procured using the Corrected Covered Area (CCA) (24). The CCA was calculated separately for each disease. Within-disease overlap was evaluated as “slight” using the standard criterial <5% slight, 5%–10% moderate, and >10% high. This was true for all three diseases: 3.6% for asthma, 2.9% for cystic fibrosis, and 1.2% for COPD. This result supported retaining all 9 reviews without overlap-based exclusion.

### 3.2 Screening of original publications

The 9 included reviews collectively referenced 231 original publications as potential sources of evidence for graph network analysis. Of these, 18 met inclusion requirements in full, while 213 were excluded. The ten most frequent exclusion reasons, in descending order, were: no generalizable or extractable barrier or facilitator reported [example: only a System Usability Scale (9) or similar Likert-based score without further qualitative content]: 83 publications; publication date before the ten-year cutoff (46 publications); no extractable evidence derived from respondent's own direct, lived experience, such as when a publication reported perceptions rather than experience, or a publication cited a healthcare professional reporting on patient's barriers and facilitators, rather than directly documenting patient's own report (29 publications). In addition, many original publications referenced by included reviews were of a wrong publication type for this umbrella review (e.g., conference proceedings, book chapters, further reviews, or doctoral thesis): 26 publications. Further exclusion reasons were unclear or irrelevant disease focus, or when a publication focused on more than one disease without disease-specific attribution of its findings (12 publications); prior identification through an already-screened reference list (12 publications); retrospective study design (2 publications); language other than English (1 publication); substantial reporting overlap with an already-included publication from the same research group (1 publication). Finally, full text of one publication could not be located.

As mentioned above, final review sample included no publication focusing on COVID-19 or Long COVID. Of 32 original publications whose titles or abstracts were selected as COVID-related, 25 were excluded for the same leading reason indicated above – no generalizable or extractable finding. The remainder of COVID literature was also excluded, in particular, predominantly because they focused on telemedicine delivered during the pandemic, rather than COVID-19 itself as the studied disease. This was despite the fact that during the extended search, five recent reviews addressing COVID-19, Long COVID, or Post-COVID digital health implementation were identified and evaluated as full texts (PMID 38594927, 41212631, 41138066, 40440284, 39269888). All five were excluded because their cited original publications did not present barriers or facilitators as findings stratified by the source publication itself, consistent with the eligibility criteria.

### 3.3 Included original publications by disease

Among 18 original publications included (Table 1), there were 8 with the focus on asthma, 6 concerning COPD, and 4 addressing CF. Comparisons of these publications revealed an interesting pattern. The asthma and CF original publications presented evidence that comprised two levels of patient care: direct reporting of individual patients' experiences or those of healthcare staff vs. published well-structured and framework-mapped experience of entire institutions or regional/national level programs. For asthma, three included original publications (25–27) comprehensively described an established school-based, pediatric asthma telehealth program, or a comparison between school participants at different stages of program implementation. A similar institution- or program-level evidence was findable in CF original publications, including a detailed program-level study that applied CFIR framework to stratify the output of a national survey administered by the US Cystic Fibrosis Foundation. In contrast, COPD original publications included in this reference sample did not present a similar two-tier evidence source. Specifically, all 6 included original publications were typical investigator-initiated studies, mostly from a single study and with a single panel of study participants.

### 3.4 Technology coverage by disease

In this reference sample of original publications, eHealth, mHealth, and telemedicine-related studies were approximately homogenously distributed across 8 asthma publications. The 6 included COPD original publications more narrowly covered technology, since 5 publications covered mHealth and 1 addressed telemedicine. All 4 CF publications focused on telemedicine applications.

### 3.5 Network findings

Graph-theoretic network representations of interconnectivity of barriers and facilitators were built using base R, R Studio, as well as appropriate packages (17–22). For granularity, the networks were built separately for patient-reported vs. healthcare staff-reported findings, both additionally separated for each disease. If two codes were reported within the same publication (e.g., independently by patients and healthcare professionals), graph algorithm joined them by a line. Correspondingly, identical codes reported by two separate publications were connected in a graph network by an additional line. The codes were color-mapped in green (facilitators), red (barriers), grey (mixed; when different sources reported the same phenomenon as both a barrier and facilitator), and navy-blue (neutral, i.e., without apparent positive or negative effect on Digital Medicine technology uptake).

The asthma-informed networks, both patient- and healthcare professional-centered, showed several distinct groups of related codes. In particular, patient-centered network demonstrated a well-structured and abundant representation of both barriers and facilitators, as well as equity dimensions (Figure 3). This finding indicated that included original publications reported richly on these factors. The richness of barrier/facilitator reporting in asthma was seen in the network that arranged the visualization of the healthcare professional-centered barriers and facilitators (Supplementary Figure S1). Crucially, there were two major sources of healthcare professional-centered inputs. In particular, three publications (25–27) provided evidence of barriers and facilitators reported surveys that evaluated regional or national school-based pediatric asthma management programs. Other publications reported barriers and facilitators from single-center studies. Correspondently, evidence from pediatric school-based asthma studies was coded separately (i.e., as “institutional” barriers and facilitators) from the healthcare professional-reported barriers and facilitators in publications from smaller-scale, single-center studies. Reflecting this, a clear separation between the barriers and facilitators was seen on network representation (Supplementary Figure S1). In particular, the evidence from school program based-publications was appropriately clustered together, while findings from other healthcare professional-reported evidence was clustered separately.

**Figure 3:**
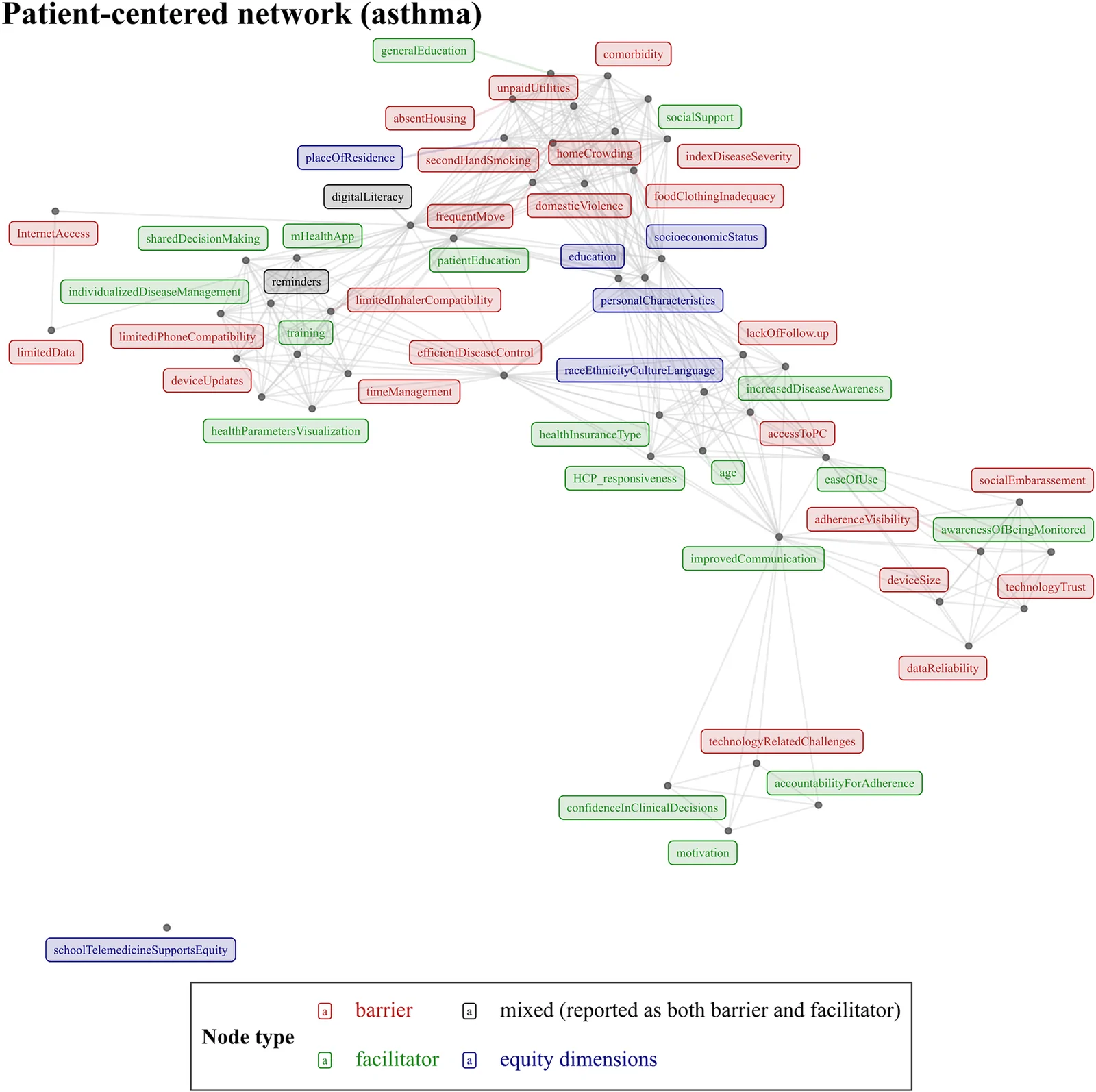
Patient-centered network (asthma). Graph-theoretic network representations of patient-centered barriers (red), facilitators (green), and equity dimensions (blue) in asthma. Labels (“nodes”) represent reported findings of included original publications, and lines (“edges”) represent connections.

For CF, the patient network was sparse, with three small, separate clusters of barriers and facilitators (Figure 4). Each cluster was drawn from a separate publication, with no connecting lines between the clusters. No healthcare professional-centered network could be built using included original publications in CF. Specifically, only one publication reported healthcare professional-level evidence, such that the network building algorithm could not pair these findings with another same source. There were also two equity-related findings from one CF publication (Figure 4). Of note, one publication in CF (28) provided an abundant list of healthcare professional-centered barriers and facilitators. This publication approached the subject of Digital Medicine as part of the survey initiated by the US CF Foundation (28). Therefore, the reported barriers and facilitators were considered by the investigators as “institutional” barriers and facilitators. Moreover, the aforementioned publication pre-structured the published results according to domains and constructs of previous version of CFIR framework (29). Based on these two considerations, this publication's findings were subjected to a dendronetwork analysis presented in a subsequent subsection.

**Figure 4:**
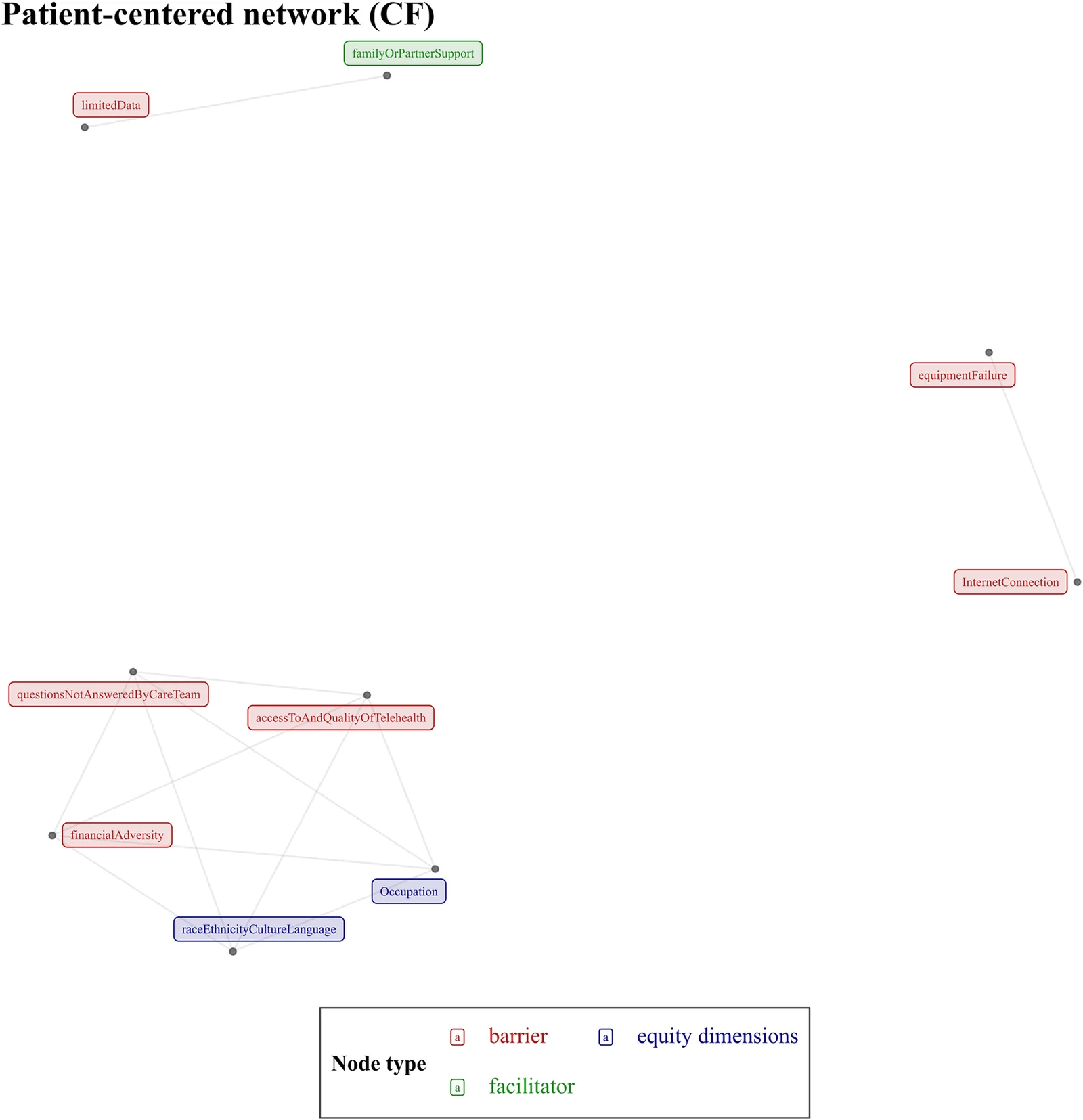
Patient-centered network (CF). Graph-theoretic network representations of patient-centered barriers (red), facilitators (green), and equity dimensions (blue) in CF. Labels (“nodes”) represent reported findings of included original publications, and lines (“edges”) represent connections.

For COPD, both the patient- and healthcare professional-centered networks (respectively, Figures 5, Supplementary Figure S2) were somewhat denser than the patient-centered CF network (Figure 4). The COPD-informed networks comprised several small, separate clusters that obviously represented individual publications, with limited connection between the clusters. Interestingly, both CF and COPD barrier and facilitator networks were dominated by barrier findings (Figures 4, 5, and Supplementary Figure S2), whereas asthma-informed networks were more balanced out with regard to proportions of barriers vs. facilitators (Figure 3).

**Figure 5:**
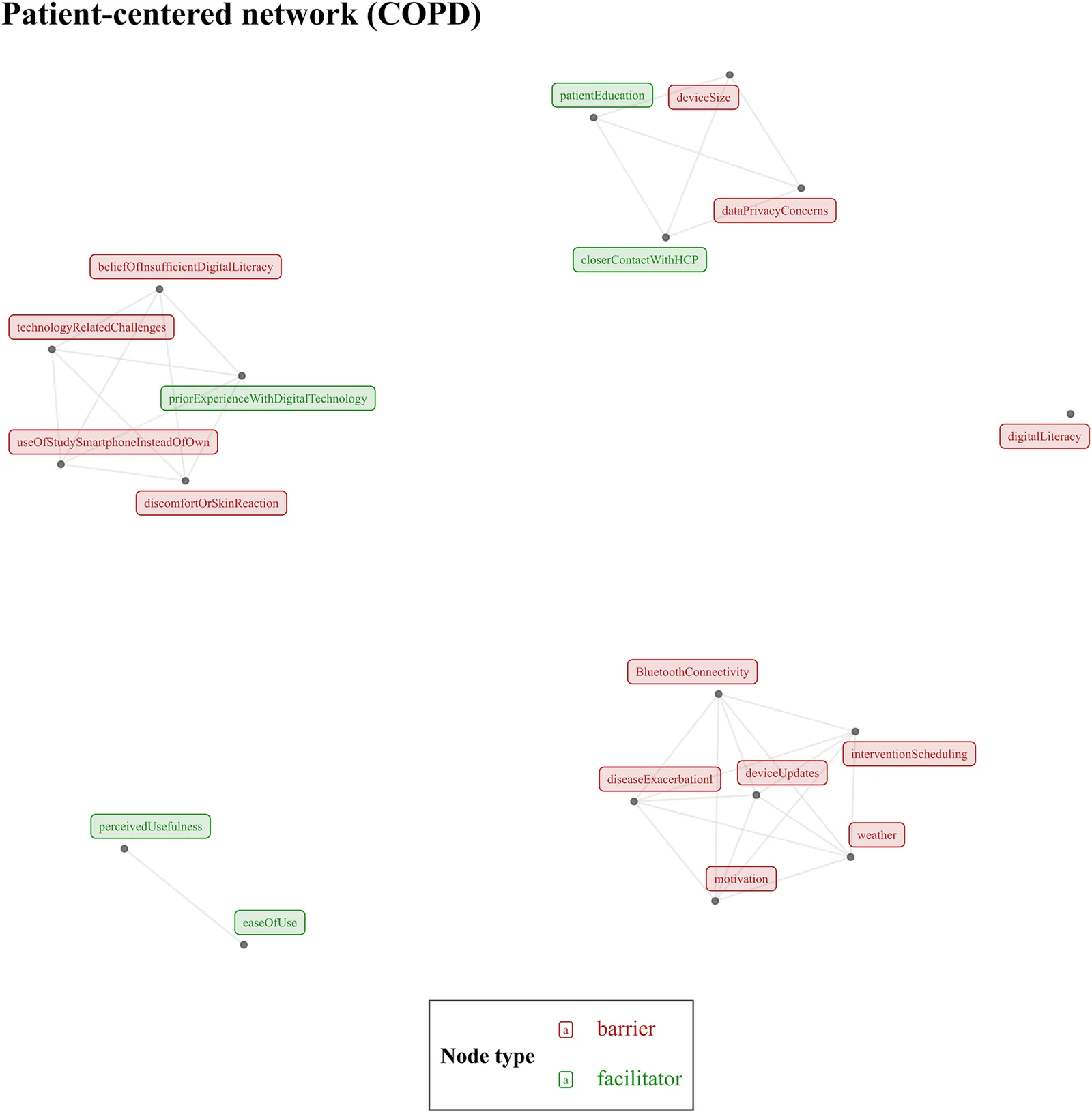
Patient-centered network (COPD). Graph-theoretic network representations of patient-centered barriers (red), facilitators (green), and equity dimensions (blue) in COPD. Labels (“nodes”) represent reported findings of included original publications, and lines (“edges”) represent connections.

Overall, across all three diseases, the structure of respective networks aligned closely with the number of contributing publications. Where several publications shared overlapping findings, as was the case in the asthma networks, the resulting figure showed real connecting structure beyond a single publication. Where only one or two publications contributed to a given node cluster, as was the case in CF and, to a lesser extent, COPD, the resulting network showed separate and well-isolated clusters with no connections between them. It was, theoretically, possible to harmonize several seemingly similar findings to a single code. The investigators put forward the following two considerations against extensive harmonization. For instance, codes like “limitediPhoneCompatibility”, “limitedInhalerCompatibility”, “BluetoothConnectivity”, or “deviceUpdates” – all reported as barriers – could have been harmonized as a single “technologyRelatedChallenges”, fostering higher interconnectivity. However, extraction of barriers and facilitators to Digital Medicine in both CF and COPD did not form rich basis for such harmonization. At the end, harmonization might have prevented the employed algorithmic network building. Second consideration was that planned mapping to overarching domains of a framework would be more informative in identification commonalities and differences across the three diseases.

### 3.6 Mapping of reported CF barriers and facilitators onto the CFIR framework

The previously mentioned CF publication (28) was subjected to dendronetwork analysis because the reported evidence had sufficient variety and depth to allow a full check against nearly every construct of the CFIR framework [previous version (29)]. Crucially, this publication collected separate data from two groups of program clinics: (Group A) clinics that rated telemedicine as similar in quality to, or even better, than in-person care, and subsequently, already implemented telemedicine in routine care, and (Group B) clinics that rated telemedicine worse that in-person care and had a slower uptake. This stratification permitted to visualize both findings as separate dendronetworks. Additional methodological details are presented in Supplementary Materials.

Four constructs from the 2009 CFIR framework (29) were never reported by this publication (28), and several other constructs were reported for only one group of clinics. The investigators wanted to retain these gaps visible in the dendronetwork and assigned separate colors to missing or partially reported constructs. Re-mapping this publication's findings onto the 2022 version of the CFIR framework (2) was not feasible from the available data extracted from the publication. In particular, current CFIR framework structure has substantial differences from the 2009 version, which precluded revised mapping.

The findings of healthcare professional-centered, CFIR-mapped barriers and facilitators, stratified by the aforementioned Group A and B, are presented, respectively, in Figures 6, 7. Firstly, these Figures demonstrate principal feasibility of building a hierarchical (dendronetwork) network visualization of barriers and facilitators. Obviously, the prerequisite is that evidence collection by an underlying study was informed by a construct-reach investigative framework. Second, these Figures respectively demonstrate how the same factor may be viewed in different polarities (i.e., as a facilitator or barrier) by different groups of healthcare professionals. Finally, evidence visualization in these Figures confirm that a framework-mapped network of barriers and facilitators is very helpful for instant identification of the underlying trends, as well as knowledge gaps. For instance, a combination of larger bubble sizes and green color – alone or in combination – are intuitively associated with positive trends. Conversely, larger bubble sizes that are shaded orange or red are instantly understood as a substantial negative trend. Motivated by these observations, the investigators next mapped all findings from across all included original publications to the Chaudoir et al. framework (1).

**Figure 6:**
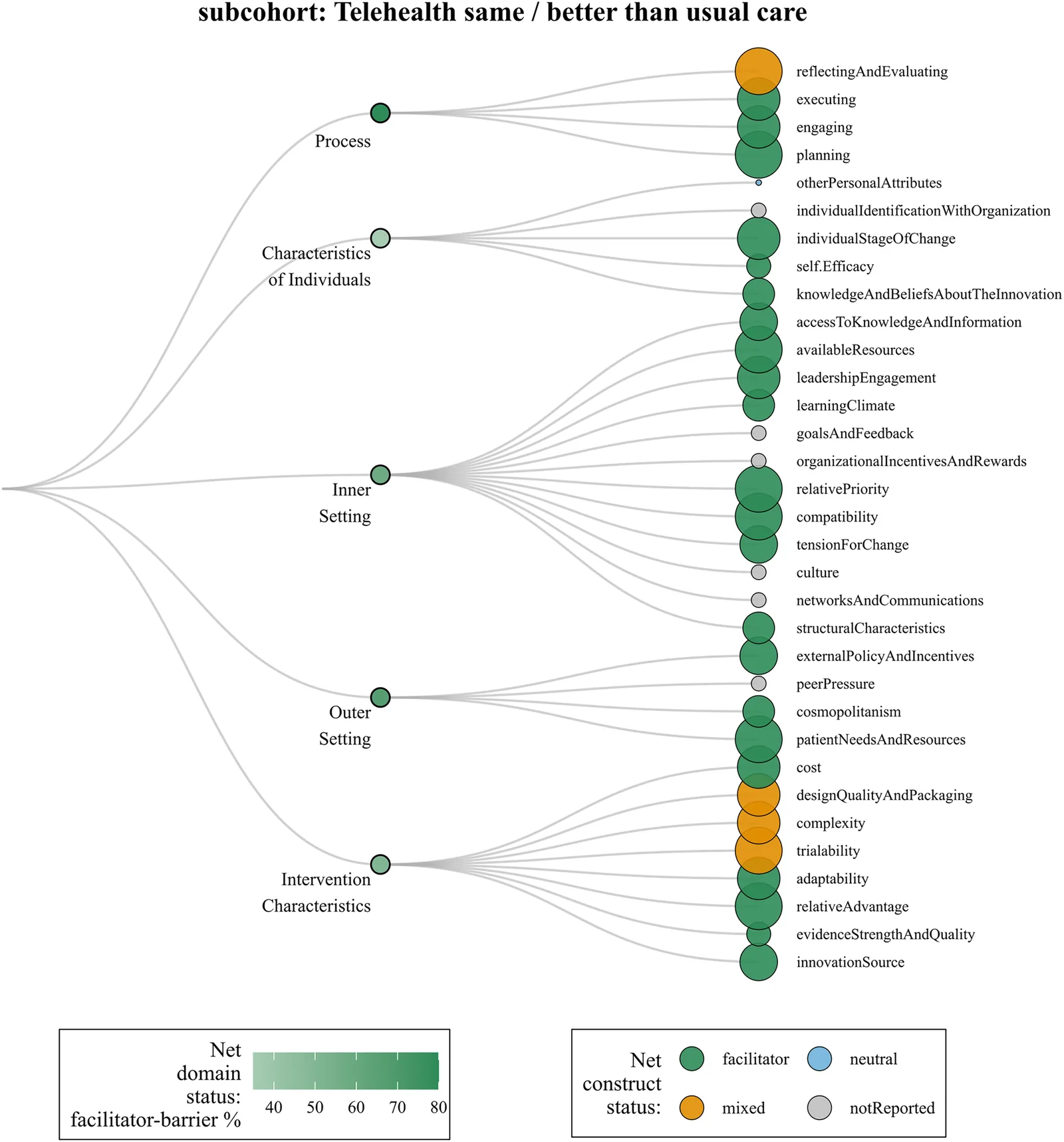
(Group A) Dendronetwork representation of healthcare professional-centered, institutional facilitators (green), mixed factors (reported by some clinics as a barrier and by other clinics as a facilitator; orange), and neutral contributors (blue), mapped to constructs of the 2009 CFIR framework. Clinics within Group A did not report barriers consistently, leading to the absence of constructs in red color. Construct sizes reflect the proportion of clinics that gave a construct any rating at all, regardless of polarity (i.e., facilitator, barrier, or neutral). Constructs that were never reported were visualized at a preset “void” size (<50% of minimal proportion). The five domains (“Intervention Characteristics”, “Outer Setting”, “Inner Setting”, “Characteristics of Individuals”, and “Process”) of Labels (“nodes”) aggregate the aforementioned constructs. The resulting “net domain status” is represented by a color gradient that is proportional to the average proportion (i.e., across all domain constructs) of facilitators less the proportion of barrier, excluding neutral ratings. The lines (“edges”) represent connections.

**Figure 7:**
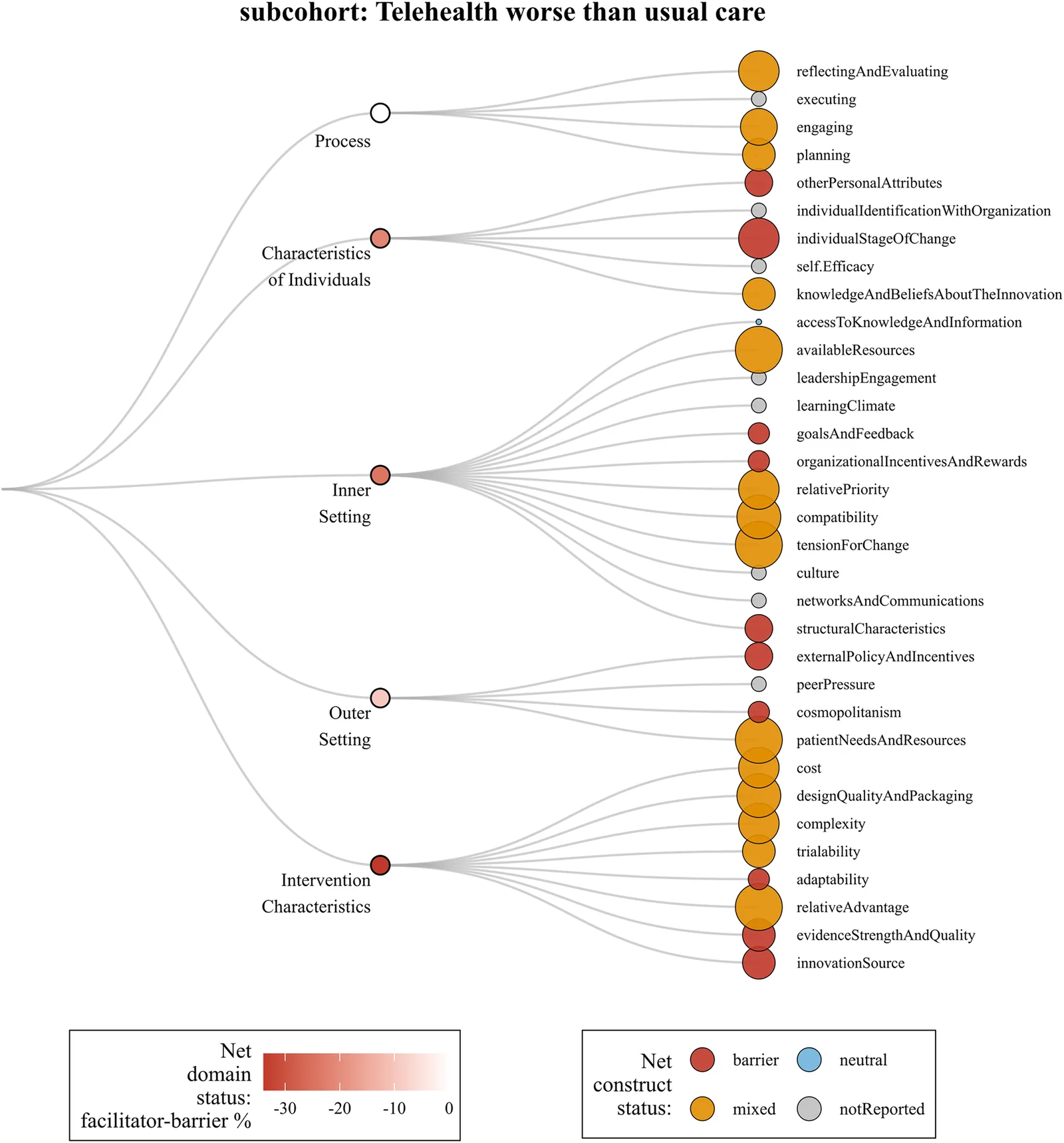
(Group B) Dendronetwork representation of healthcare professional-centered, institutional barriers (red), and mixed factors (reported by some clinics as a barrier and by other clinics as a facilitator; orange), and neutral contributors (blue), mapped to constructs of the 2009 CFIR framework. Clinics within Group B rarely reported facilitators, leading to the absence of constructs in green color. Construct sizes reflect the proportion of clinics that gave a construct any rating at all, regardless of polarity (i.e., facilitator, barrier, or neutral). Constructs that were never reported were visualized at a preset “void” size (<50% of minimal proportion). The five domains (“Intervention Characteristics”, “Outer Setting”, “Inner Setting”, “Characteristics of Individuals”, and “Process”) of Labels (“nodes”) aggregate the aforementioned constructs. The resulting “net domain status” is represented by a color gradient that is proportional to the average proportion (i.e., across all domain constructs) of facilitators less the proportion of barrier, excluding neutral ratings. The lines (“edges”) represent connections.

### 3.7 Mapping of all reported barriers and facilitators onto the Chaudoir et al. framework

While this framework (1) is simpler than both versions of CFIR (2, 29), it combines the domains that are essential to Digital Medicine: innovation (technology), patients, healthcare providers, institutional programs, and structural support (e.g., policymaking). Table 2 summarizes the results of mapping (i.e., structured distribution) of the findings on barriers and facilitators to appropriate domain of the Chaudoir et al. framework.

**Table 2:** Mapping of reported barriers and facilitators to chaudoir et al. framework (1).

Domain	Polarity	Asthma publications (*n* = 8)	CF publications (*n* = 4)	COPD publications (*n* = 6)	Total
Technology/innovation		13^b^	8	1	22
Patient	barriers	23	5^c^	8^d^	36
	facilitators	23	1	5	29
	neutral	10	2	2	14
Provider	barriers	4	0	8	12
	facilitators	16	0	4	20
	neutral	0	0	0	0
Institutional	barriers	20	1	1^a^	22
	facilitators	10	1	0^a^	11
Structural		2	1	0	3
**Total**		**121**	**11**	**37**	**169**

Counts represent “finding-mentions”, meaning that each barrier, facilitator, or other coded factor was counted once per publication reporting it, without harmonizing across publications (i.e., two reports of “digital literacy” as a barrier result in a count of 2).

a Additional CF-informed rich institutional findings are presented as dendronetwork figures. Examples of disease-specific barriers and facilitators.

b “limited inhaler compatibility”.

c “questions not answered by care team”.

d “device size”.

In principal, this Table demonstrates that a *post-hoc* framework mapping of identified barriers, facilitators, and other factors is feasible. On a concrete level, this Table demonstrates that asthma-informed findings – corroborating the graph networks (Figures 3 and Supplementary Figure S1) – were richer across all categories in comparison to other two diseases. Moreover, even when the counts from both CF and COPD are added, the richness of asthma-informed findings could not be matched (Table 3).

**Table 3:** Mapping of reported barriers and facilitators to diseases.

Category	Barriers	Facilitators
A. Asthma		
Patient (subjective actors)	food clothing inadequacy; social embarrassment; technology trust; adherence visibility	improved communication; ease of use; increased disease awareness; awareness of being monitored; motivation; accountability for adherence; confidence in clinical decisions
Patient (objective factors)	domestic violence; index disease severity; comorbidity; home crowding; absent housing; unpaid utilities; frequent move; second hand smoking; efficient disease control; lack of follow up; digital literacy; reminders; time management; data reliability; limited data; internet access	general education; digital literacy; social support; patient education; healthcare professional responsiveness; age; health insurance type; training; health parameters visualization; reminders; m health app; individualized disease management; shared decision making
Patient (technological factors)	access to PC; device updates; limited iPhone compatibility; limited inhaler compatibility; device size; technology related challenges; limited data; internet access	*[none reported]*
Healthcare professional (subjective factors)	*[none reported]*	improved communication; ease of use; increased disease awareness; patient appreciation; improved disease management
Healthcare professional (objective factors)	workflow coordination; lack of training; time management; increased workload	workflow coordination; training; patient compliance; medication use tracking; substantiated medical decision; individualized disease management; time allocation; multidisciplinary approach; improved adherence
Healthcare professional (technological factors)	integration with EHR; computer internet access; technology related challenges	video assessment of symptoms
Institutional	training; time for training; lack of objective exam; workflow coordination; dedicated personnel or infrastructure; community resources and access; source demand and advocacy; priorities; resources and technology; school need; leadership teamwork; attitudes; school nurse competing demands; lack of family or caregiver involvement; personnel time resource allocation; budget; lack of punctuality by supporting structures; knowledge availability; internet	workflow coordination; dedicated personnel; training; time for training; synchronous monitoring; videoconsultation; sufficient funding; perseverance; leadership; organizational readiness
B. Cystic Fibrosis		
Patient (subjective factors)	equipment failure	family or partner support
Patient (objective factors)	questions not answered by care team; financial adversity; access to and quality of telehealth; internet connection	*[none reported]*
Patient (technological factors)	limited data	*[none reported]*
Healthcare professional (subjective)	*[none reported]*	*[none reported]*
Healthcare professional (objective)	*[none reported]*	*[none reported]*
Healthcare professional (technological factors)	*[none reported]*	*[none reported]*
Institutional	*[none reported]*	*[none reported]*
C. COPD		
Patient (subjective factors)	belief of insufficient digital literacy; motivation; data privacy concerns	ease of use; perceived usefulness; patient education; closer contact with healthcare professional
Patient (objective factors)	digital literacy; discomfort or skin reaction; disease exacerbation; weather; intervention scheduling	prior experience with digital technology
Patient (technological factors)	technology related challenges; use of study smartphone instead of own; Bluetooth connectivity; device updates; device size	*[none reported]*
Healthcare professional (subjective factors)	lack of training in adaptation to telemedicine; limited exercise variation	joint decision making in patient selection; initial offline meeting
Healthcare professional (objective factors)	time management; increased workload; workflow coordination; difficulty to adapt existing protocols to new technology; difficulty to adapt new technology into existing protocols	close collaboration within team; prioritization of telemedicine by superiors
Healthcare professional (technological factors)	technology related challenges; data veracity	*[none reported]*
Institutional	lack of personnel interoperability	*[none reported]*

### 3.8 Identified transferrable concepts and recommendations

The investigators deemed the following ideas and findings as transferrable concepts with high relevance to fostering the implementability of Digital Medicine. First, health and equity dimensions can be appropriately addressed even in the resource-strapped settings (30), when the resources available in the public (e.g., public libraries, healthcare centers) are utilized to promote the equality in the access to Digital Medicine (30). Second, while the field of barrier/facilitator appraisal continue to predominantly utilize qualitative and mixed-methods approaches, there are promising trends toward integration of quantitative outputs. For instance, included publications utilized quantitative metrics to distinguish between technology adoption vs. “sustained use” (31). When this approach was applied to several variables, it fostered building predictive statistical models of technology utilization (31). Third, simultaneous technology evaluation in different groups of end users, in particular, “utilizers” vs. “non-utilizers” (26, 32, 33) enables utilization of a randomized controlled trial format (30, 33–35). Fourth, several included publications prospectively utilized established frameworks, which fostered procurement of rich and comprehensive evidence (27, 28, 31, 32). Finally, prospective planning of comprehensive assessments of barriers and facilitators, as well as a subsequent generalizable recommendations output, is therefore advisable. A comprehensive example of such generalizable recommendations can be found in one included original publication (32).

## 4 Discussion

This umbrella review had an objective to identify common barriers, facilitators, and transferable concepts for Digital Medicine. With additional focus on Respiratory Medicine, bronchial asthma, CF, COPD, COVID-19 served as examples of respiratory diseases whose prevalence in the general population ranges from the high to low, or fluctuates during and after the pandemic. These four diseases formed the “Population” search concept of this review, while “Intervention” comprised diverse technologies of Digital Medicine: mHealth, remote digital monitoring, ePROM collection, and telemedicine. To ensure high fidelity of extracted evidence, each identified review publication was subjected to additional screening of original publications that were referenced within the former. Only review publications that withstand this additional scrutiny were included. Similarly, only original publications that reported clearly attributable barriers and facilitators identified through own experience (as opposed to perceptions or attitudes) became part of the final reference sample for extraction of barriers and facilitators. Of note, this review was conceptualized as an exploratory tool to enable evidence extraction across four diseases and several Digital Medicine technologies. Given the scope of searched literature, direct search and identification of original publications across four diseases (especially with inclusion of COVID-19), combined with data preparations for granular network analysis, would have required a substantially higher volume of work. This investment could have been justified only if the applied strategy is proven successful, which is the current view that the investigators hold of this project and the analytical pipeline at its core.

Crucially, though, one of the clearest answers to investigators' research question was procured not through an evidence synthesis using included review publications. This finding was only feasible because of the employed two-step screening procedure that scrutinized both the identified review publications and their references as the source of evidence.

This statement does not aim to undermine the quality of screened review publications. Importantly, included reviews demonstrated the fidelity of their evidence synthesis by “surviving” the double-step appraisal applied in the analytical pipeline described above. Furthermore, it should be acknowledged that the majority of excluded review publications also had high fidelity of evidence synthesis. The reasons for exclusion were as often as trivial as the focus on e.g., perceptions towards Digital Medicine or behavioral patterns, instead of actual experiences.

Nonetheless, identification of the aforementioned original CF publication (28) was crucial because it had already structured its findings around the most popular CFIR implementation framework [2009 version (29)]. Furthermore, this publication's findings were mapped onto CFIR constructs with high fidelity and completeness. Additional original publications in pediatric asthma that are included in this umbrella review (27) further extended this trend. In particular, the findings in these original publications could be extracted, classified, and mapped onto established frameworks with sufficient depth.

By comparison, other screened review and original publications – while offering valuable and high-quality evidence – were less comprehensive in reporting barriers and facilitators to Digital Medicine implementation. More often than not, technology acceptance was appraised with help of validated tools that did not mean to comprehensively assess subjective and objective factors in a study sample that comprises patients, their families, healthcare practitioners, and other stakeholders. This finding has a practical implication for future Digital Medicine research. A study that appraises technology acceptance only through a usability score [e.g., the System Usability Scale (9)] cannot later be mapped onto an implementation framework with sufficient veracity. In contrast, a study that includes a qualitative or mixed-methods substudy that is prospectively built on the basis of an established framework avoids such problem.

A second key finding of this umbrella review came from original publications that reported findings from the US school-based pediatric asthma patient programs, as well as single-center, asthma-related studies (26, 27, 30, 31). Several equity-related items, including place of residence and level of education, were reported in addition to digital literacy (a stereotypical Digital Medicine barrier) within the same publications. As several publications in this reference sample report pediatric findings, asthma patient population is considerably younger. Therefore, digital literacy should not play a crucial role in contrast to older COPD patients. However, this umbrella review identified additional factors that regardless of digital literacy restrict an individual uptake of digital technologies. These factors included income, language, adequacy of available Internet or mobile connections. Moreover, support – or lack thereof – by families/caregivers was independently identified in both pediatric and adult asthma studies as a prominent facilitator (or barrier (26, 30);.

Third important finding is that COPD publications are sufficiently abundant in numbers and diverse in scope. Yet the evidence on barriers and facilitators – at least in this reference sample – is comparably less rich than in asthma publications. In the CF field, a similar “data scarcity” was made up by the availability of well-structured, comprehensive, and framework-informed assessments of barriers and facilitators to Digital Medicine [e.g. (28),]. Until similar institutional programs are introduced for COPD, individual studies should take into consideration good examples of comprehensive and multifaceted approaches to barrier and facilitator assessment. A better balanced approach will overcome current asymmetry of the evidence on barriers and facilitators identified in COPD publications (Figure 5). Proper appreciation of facilitators to Digital Medicine can help devise efficient practical actions. For example, this umbrella review identified some interesting combinations: “prior experience with digital technology” vs. “patient education” and “closer contact with HCP” (Figure 5). Subsequent Digital Medicine-promoting initiatives in COPD may be structured around these facilitators, specifically aiming to bridge the presently separated facilitators (Figure 5). For example, Digital Medicine uptake may be reinforced by repeated training sessions that gradually increase exposure, with occasional progress verification jointly with a healthcare professional. While requiring time and personnel commitment, such programs would have a payback in increased digital engagement of a COPD patient. This, in turn, would reinforce the uptake of remote home rehabilitation and digital exacerbation monitoring programs, which are both crucial for COPD.

The transferable concepts identified in Results are presented as a list rather than a single, unified synthesis or framework. Since 18 included publications spread across three diseases and different Digital Medicine technologies, it would not be realistic to develop a realistic unified synthesis. Subsequent systematic reviews should address the identified themes, concepts, and recommendations in depth.

It would have been interesting to stratify barriers and limitations also by technology type in addition to this report's disease-based stratification. Such analysis could procure valuable insights. After deliberation, the investigators did not pursue this idea. As aggregated data in this report demonstrate, telemedicine approaches were found almost exclusively in CF-related publications. In contrast, mHealth applications for remote monitoring or home-based pulmonary rehabilitation were presented in COPD-related publications. Furthermore, mHealth applications for smart inhalers were discussed in asthma-related publications. As a result, stratification by technology could have been confounded by the disease-driven patterns of technology use. A proper disentanglement of the disease vs. technology patterns requires collecting a substantially larger evidence base, which is a valuable direction for future studies.

Additional three limitations should also be acknowledged. First, classification of barriers and facilitators involved a degree of subjectivism, which is not foreign to any review of this type. Second, it was mentioned above that this umbrella review was designed to be exploratory. Still, covering four diseases and several technology types at once, with an overarching objective to identify new research concepts, inevitably fosters heterogeneity. The scope, therefore, comes at a cost of having an inherent limitation. Specifically, there was a necessary trade-off between the scope and depth within each studied diseases or technologies. As mentioned above, this, for instance, limited the investigators from building a unified synthesis or an overarching model. Third, while this umbrella review's search for relevant review publications ended on 31 May 2026, t the reason why the investigators did not identify review or original publications concerning AI-enabled chatbots could have been related to a structural limitation of umbrella reviews. The latter rely on availability of already published reviews, which – in turn – require a sufficient body of published original studies to accumulate. Combined, these two factors result in evidence base being somewhat behind the most current literature. This especially matters for fast-paced development in the area of Digital Medicine. The investigators therefore consider a follow-up systematic review of original publications as a valuable next step to extract and appraise the evidence, especially from publications that address the newest technology developments.

Jointly, the most consistently reported barriers across included publications were technology-related implementation challenges, time management challenges, and gaps in workflow coordination. Conversely, improved communication, structured patient education, and ease of use were the most consistently reported facilitators. For policymakers and implementers, this pattern translates into three concrete priorities: (i) investing in interoperable, low-maintenance technology and dedicated technical support to reduce device- and connectivity-related barriers; (ii) integrating Digital Medicine operations within existing clinical workflows, rather than adding them as parallel duties, to address the workflow-coordination and time-management burden reported by both patients and healthcare professionals; and (iii) prioritizing structured onboarding and patient education. Especially the latter links well with the ease of use and communication as the facilitators. Importantly, several initiatives are not tied up to a specific disease. This includes digital literacy support, which, was reported across more than one disease group, underscoring its relevance as a transferable target for policy independent of the specific respiratory condition being treated. These initiatives complement the transferable concepts and recommendations identified above. Jointly, they should facilitate translation of review's findings into actionable implementation strategy.

In summary, the present umbrella review identified high-quality, framework-mappable evidence on Digital Medicine barriers and facilitators described in included original publications. This was the most important payoff of the deployed stringent analytical pipeline that was at the core of this review's methodology. Comparisons across the studied diseases, as well as within included and excluded publications further highlight the importance of planning comprehensive and properly structured evidence that supports uptake and sustained success of Digital Medicine.

## Data Availability

The original contributions presented in the study are included in the article/Supplementary Material, further inquiries can be directed to the corresponding author.

## References

[1] Chaudoir SR Dugan AG Barr CH. Measuring factors affecting implementation of health innovations: a systematic review of structural, organizational, provider, patient, and innovation level measures. Implement Sci. (2013) 8:22. 10.1186/1748-5908-8-22 23414420 PMC3598720

[2] Damschroder LJ Reardon CM Widerquist MAO Lowery J. The updated consolidated framework for implementation research based on user feedback. Implement Sci. (2022) 17(1):75. 10.1186/s13012-022-01245-0 36309746 PMC9617234

[3] Page MJ McKenzie JE Bossuyt PM Boutron I Hoffmann TC Mulrow CD. The PRISMA 2020 statement: an updated guideline for reporting systematic reviews. Br Med J. (2021) 372:n71. 10.1136/bmj.n71 PMC8005924 33782057

[4] Team TE. EndNote. Philadelphia, PA: Clarivate (2013). p. EndNote 20.

[5] Bramer WM Giustini D De Jonge GB Holland L Bekhuis T. De-duplication of database search results for systematic reviews in EndNote. J Med Libr Assoc. (2016) 104(3):240–3. 10.3163/1536-5050.104.3.014 27366130 PMC4915647

[6] Ouzzani M Hammady H Fedorowicz Z Elmagarmid A. Rayyan-a web and mobile app for systematic reviews. Syst Rev. (2016) 5(1):210. 10.1186/s13643-016-0384-4 27919275 PMC5139140

[7] Dauletbaev N Oftring ZS Akik W Michaelis-Braun L Korel J Lands LC. A scoping review of mHealth monitoring of pediatric bronchial asthma before and during COVID-19 pandemic. Paediatr Respir Rev. (2022) 43:67–77. 10.1016/j.prrv.2022.01.002 35131174 PMC8761580

[8] Shea BJ Reeves BC Wells G Thuku M Hamel C Moran J. AMSTAR 2: a critical appraisal tool for systematic reviews that include randomised or non-randomised studies of healthcare interventions, or both. Br Med J. (2017) 358:j4008. 10.1136/bmj.j4008 PMC5833365 28935701

[9] Brooke J. SUS—a quick and dirty usability scale. In Jordan PW Thomas B Weerdmeester BA McClelland IL, editors. Usability Evaluation in Industry. London: Taylor & Francis (1996). p. 189–194.

[10] Mangion A Piller N. What is digital health, eHealth, uHealth and healthcare 4.0? Health Informatics. (2025) 2(2):137–42. 10.1016/j.infoh.2025.06.002

[11] Kelly JT Campbell KL Gong E Scuffham P. The internet of things: impact and implications for health care delivery. J Med Internet Res. (2020) 22(11):e20135. 10.2196/20135 33170132 PMC7685921

[12] Vaezi A Ashby T Kadir S Schweitzer M Hadeh A Sen P. Defining physician-AI collaboration in pulmonary and critical care medicine: concepts and illustrative examples for clinical reasoning. ATS Sch. (2026) 7(2):97–102. 10.1093/atsscholar/aapag001 41721782 PMC13220959

[13] Davis FD. Perceived usefulness, perceived ease of use, and user acceptance of information technology. MIS Q. (1989) 13(3):319–40. 10.2307/249008

[14] Gagnon MP Orruño E Asua J Abdeljelil AB Emparanza J. Using a modified technology acceptance model to evaluate healthcare professionals’ adoption of a new telemonitoring system. Telemed J E Health. (2012) 18(1):54–9. 10.1089/tmj.2011.0066 22082108 PMC3270047

[15] Michie S Van Stralen MM West R. The behaviour change wheel: a new method for characterising and designing behaviour change interventions. Implement Sci. (2011) 6:42. 10.1186/1748-5908-6-42 21513547 PMC3096582

[16] Kitson A Harvey G McCormack B. Enabling the implementation of evidence based practice: a conceptual framework. Qual Health Care. (1998) 7(3):149–58. 10.1136/qshc.7.3.149 10185141 PMC2483604

[17] R Core Team. R: A language and environment for statistical computing. R Foundation for Statistical Computing, Vienna, Austria. version 4.5.0 (2025-04-11) ed. (2025). Available online at: https://www.R-project.org/2025 (Accessed April 4, 2025).

[18] RStudio Team. RStudio: Integrated Development for R. RStudio, PBC, Boston, MA. Available online at: http://www.rstudio.com/ (Accessed April 4, 2025).

[19] Csárdi G Nepusz T. The igraph software package for complex network research. InterJ. Complex Syst. (2006) 1695. Available online at: https://igraph.org.

[20] Pedersen TL. ggraph: An implementation of grammar of graphics for graphs and networks. R package version 2.1.0. (2023). 10.32614/cran.package.ggraph. version 2.1.0.

[21] Pedersen T. tidygraph: A tidy API for graph manipulation. R package version 1.2.3. (2022). Available online at: https://github.com/thomasp85/tidygraph.

[22] Wickham H. ggplot2. Elegant Graphics for Data Analysis. New York, NY: Springer-Verlag (2016).

[23] Bougioukas K Pamporis K Karakasis P. amstar2Vis: Plots the AMSTAR barplot. R package version 0.1.0, commit 421e3bdb90d256edccdcd43b94cf5a55e54c2d37. Available online at: https://github.com/bougioukas/amstar2Vis2026 (Accessed April 4, 2025).

[24] Hennessy EA Johnson BT. Examining overlap of included studies in meta-reviews: guidance for using the corrected covered area index. Res Synth Methods. (2020) 11(1):134–45. 10.1002/jrsm.1390 31823513 PMC8555740

[25] Johnson EE MacGeorge C Andrews A King KL Teufel RJ Brinton DL. Nurse perspectives regarding implementation of an asthma monitoring mobile health application in the school setting. Telemed J E Health. (2021) 27(8):955–62. 10.1089/tmj.2021.0100 34152858 PMC8432601

[26] MacGeorge CA King K Andrews AL Sterba K Johnson E Brinton DL. School nurse perception of asthma care in school-based telehealth. J Asthma. (2022) 59(6):1248–55. 10.1080/02770903.2021.1904978 33730979

[27] Johnson EE MacGeorge C King KL Andrews AL Teufel RJ Kruis R. Facilitators and barriers to implementation of school-based telehealth asthma care: program champion perspectives. Acad Pediatr. (2021) 21(7):1262–72. 10.1016/j.acap.2021.04.025 33940203

[28] Van Citters AD Dieni O Scalia P Dowd C Sabadosa KA Fliege JD. Barriers and facilitators to implementing telehealth services during the COVID-19 pandemic: a qualitative analysis of interviews with cystic fibrosis care team members. J Cyst Fibros. (2021) 20(3):23–8. 10.1016/j.jcf.2021.09.004 34930537 PMC8683122

[29] Damschroder LJ Aron DC Keith RE Kirsh SR Alexander JA Lowery JC. Fostering implementation of health services research findings into practice: a consolidated framework for advancing implementation science. Implement Sci. (2009) 4(1):50. 10.1186/1748-5908-4-50 19664226 PMC2736161

[30] Apter AJ Localio AR Morales KH Han X Perez L Mullen AN. Home visits for uncontrolled asthma among low-income adults with patient portal access. J Allergy Clin Immunol. (2019) 144(3):846–53.e11. 10.1016/j.jaci.2019.05.030 31181221 PMC6742549

[31] Fiks AG DuRivage N Mayne SL Finch S Ross ME Giacomini K. Adoption of a portal for the primary care management of pediatric asthma: a mixed-methods implementation study. J Med Internet Res. (2016) 18(6):e172. 10.2196/jmir.5610 27357835 PMC4945817

[32] Van Der Kamp M Reimering Hartgerink P Driessen J Thio B Hermens H Tabak M. Feasibility, efficacy, and efficiency of eHealth-supported pediatric asthma care: six-month quasi-experimental single-arm pretest-posttest study. JMIR Form Res. (2021) 5(7):e24634. 10.2196/24634 34309568 PMC8367169

[33] Adejumo I Patel M McKeever TM Shaw DE Bains M. Qualitative study of user perspectives and experiences of digital inhaler technology. NPJ Prim Care Respir Med. (2022) 32(1):57. 10.1038/s41533-022-00320-9 36550136 PMC9780314

[34] Ronan P Mian A Carr SB Madge SL Lorenc A Robinson N. Learning to breathe with Tai Chi online - qualitative data from a randomized controlled feasibility study of patients with cystic fibrosis. Eur J Integr Med. (2020) 40:101229. 10.1016/j.eujim.2020.101229 33106755 PMC7578181

[35] Bentley CL Powell L Potter S Parker J Mountain GA Bartlett YK. The use of a smartphone app and an activity tracker to promote physical activity in the management of chronic obstructive pulmonary disease: randomized controlled feasibility study. JMIR Mhealth Uhealth. (2020) 8(6):e16203. 10.2196/16203 32490838 PMC7301262

[36] Uzzaman N Hammersley V McClatchey K Sheringham J Singh D Habib GMM. Effectiveness and acceptability of asynchronous digital health in asthma care: mixed methods systematic review. J Med Internet Res. (2024) 26:e57708. 10.2196/57708 39626243 PMC11653041

[37] Hakizimana A Devani P Gaillard EA. Current technological advancement in asthma care. Expert Rev Respir Med. (2024) 18(7):499–512. 10.1080/17476348.2024.2380067 38992946

[38] Lawrie L Turner S Cotton SC Wood J Morgan HM. A qualitative process evaluation within a clinical trial that used healthcare technologies for children with asthma-insights and implications. PLoS One. (2023) 18(1):e0280086. 10.1371/journal.pone.0280086 36603013 PMC9815588

[39] Chike-Harris KE Miller S Nichols M McElligott J Kelechi T. The management of pediatric asthma using telehealth: an integrative review. Telemed J E Health. (2024) 30(3):609–21. 10.1089/tmj.2023.0109 37624652

[40] Vagg T Shanthikumar S Ibrahim H O'Regan P Chapman WW Kirwan L. Telehealth in cystic fibrosis. A systematic review incorporating a novel scoring system and expert weighting to identify a ‘top 10 manuscripts’ to inform future best practices implementation. J Cyst Fibros. (2023) 22(4):598–606. 10.1016/j.jcf.2023.05.012 37230808 PMC10204901

[41] Peckham D Spoletini G. Impact of digital technologies on clinical care for adults with cystic fibrosis. Semin Respir Crit Care Med. (2023) 44(2):217–24. 10.1055/s-0042-1758730 36535666

[42] Albon D Van Citters AD Ong T Dieni O Dowd C Willis A. Telehealth use in cystic fibrosis during COVID-19: association with race, ethnicity, and socioeconomic factors. J Cyst Fibros. (2021) 20(3):49–54. 10.1016/j.jcf.2021.09.006 34930543 PMC8683127

[43] Yap T Jones A. The feasibility and efficacy of telespirometry for pulmonary monitoring of cystic fibrosis: a systematic review. Internet J Allied Health Sci Pract. (2022) 20:Article 20. 10.46743/1540-580X/2022.2165

[44] Logie K Welsh L Ranganathan SC. Telehealth spirometry for children with cystic fibrosis. Arch Dis Child. (2020) 105(12):1203–5. 10.1136/archdischild-2019-317147 31694805

[45] O'Connor Ú Crilly G. The experiences and perceptions of telehealth in patients living with advanced chronic obstructive pulmonary disease: a qualitative evidence synthesis. J Adv Nurs. (2025) 81(4):1700–16. 10.1111/jan.16493 PMC11896827 39420778

[46] van Lieshout F Yang R Stamenova V Agarwal P Cornejo Palma D Sidhu A. Evaluating the implementation of a remote-monitoring program for chronic obstructive pulmonary disease: qualitative methods from a service design perspective. J Med Internet Res. (2020) 22(10):e18148. 10.2196/18148 33034565 PMC7584983

[47] Ramachandran HJ Oh JL Cheong YK Jiang Y Teo JYC Seah CWA. Barriers and facilitators to the adoption of digital health interventions for COPD management: a scoping review. Heart Lung. (2023) 59:117–27. 10.1016/j.hrtlng.2023.02.004 36801546

[48] Damhus CS Emme C Hansen H. Barriers and enablers of COPD telerehabilitation—a frontline staff perspective. Int J Chron Obstruct Pulmon Dis. (2018) 13:2473–82. 10.2147/COPD.S167501 30154650 PMC6103610

[49] Knox L Gemine R Rees S Bowen S Groom P Taylor D. Using the technology acceptance model to conceptualise experiences of the usability and acceptability of a self-management app (COPD.Pal®) for chronic obstructive pulmonary disease. Health Technol (Berl). (2021) 11(1):111–7. 10.1007/s12553-020-00494-7 33262925 PMC7690946

[50] Wilde LJ Sewell L Percy C Ward G Clark C. What are the experiences of people with COPD using activity monitors?: a qualitative scoping review. Copd. (2022) 19(1):88–98. 10.1080/15412555.2022.2033192 35132933

[51] Loeckx M Rabinovich RA Demeyer H Louvaris Z Tanner R Rubio N. Smartphone-based physical activity telecoaching in chronic obstructive pulmonary disease: mixed-methods study on patient experiences and lessons for implementation. JMIR Mhealth Uhealth. (2018) 6(12):e200. 10.2196/mhealth.9774 30578215 PMC6320438

[52] Wu R Liaqat D De Lara E Son T Rudzicz F Alshaer H. Feasibility of using a smartwatch to intensively monitor patients with chronic obstructive pulmonary disease: prospective cohort study. JMIR Mhealth Uhealth. (2018) 6(6):e10046. 10.2196/10046 29903700 PMC6024103

