# Supplementary material for: Common barriers and facilitators to the implementation of digital medicine in chronic respiratory diseases: a systematic review of reviews
Source: Front Digit Health. 2026 Oct 26;8:1927835. doi: 10.3389/fdgth.2026.1927835 (PMC13745709; doi:10.3389/fdgth.2026.1927835)
Supplement: Supplementary file 1 [file Datasheet1.docx]

Supplementary Material

# 1 Supplementary Figures and Tables

## 1.1 Supplementary Figures

**Supplementary Figure 1.** **Healthcare professional-centered network (asthma)**

**Figure Legend.** Graph-theoretic network representations of healthcare professional barriers (red) and facilitators (green) in asthma. Labels (“nodes”) represent reported findings of included original publications, and lines (“edges”) represent connections.

**Supplementary Figure 2.** **Healthcare professional-centered network (COPD)**

**Figure Legend.** Graph-theoretic network representations of healthcare professional barriers (red) and facilitators (green) in COPD. Labels (“nodes”) represent reported findings of included original publications, and lines (“edges”) represent connections.

## 1.2 Supplementary Tables

## Table S1A. Example of PubMed initial search strategy: bronchial asthma

1. (Asthma[MeSH Terms]) OR (Bronchial Hyperreactivity[MeSH Terms]) OR (bronchial spasm[MeSH Terms]) OR (Respiratory Hypersensitivity[MeSH Terms])

2. (asthma*[Title/Abstract]) OR (bronchial hyperreact*[Title/Abstract]) OR (bronchial hyperrespons*[Title/Abstract]) OR (Bronchoconstrict*[Title/Abstract]) OR (Bronchospas*[Title/Abstract])

3. #1 or #2

4. (Telemedicine[MeSH Terms]) OR (Remote Consultation[MeSH Terms]) OR (self care[MeSH Terms]) OR (smartphone[MeSH Terms]) OR (Mobile Applications[MeSH Terms]) OR (Wearable Electronic Devices[MeSH Terms]) OR (Internet of Things[MeSH Terms]) OR (Digital Health[MeSH Terms]) OR (Biomedical Technology[MeSH Terms]) OR (Remote Sensing Technology[MeSH Terms]) OR (Wireless Technology[MeSH Terms])

5. (telemedic*[Title/Abstract]) OR (“mobile health*”[Title/Abstract]) OR (mhealth*[Title/Abstract]) OR (telehealth*[Title/Abstract]) OR (“remote patient monitor*”[Title/Abstract]) OR (“remote monitor*”[Title/Abstract]) OR (teleconsultation*[Title/Abstract]) OR (smartphone*[Title/Abstract]) OR (wearable*[Title/Abstract]) OR (unwearable*[Title/Abstract]) OR (“mobile app*”[Title/Abstract]) OR (app[Title/Abstract]) OR (“digital health*”[Title/Abstract]) OR (“internet of thing*”[Title/Abstract]) OR (IoT[Title/Abstract]) OR (“internet of medical thing*”[Title/Abstract]) OR (“IoMT”[Title/Abstract]) OR (wireless[Title/Abstract])

6. #4 OR #5

7. (implementation science[MeSH Terms]) OR (technology assessment, biomedical[MeSH Terms])

8. (“implement*”[Title/Abstract]) OR (“facilitator*”[Title/Abstract]) OR (“enabl*”[Title/Abstract]) OR (“barrier*”[Title/Abstract]) OR (“added value*”[Title/Abstract]) OR (“technology acceptance*”[Title/Abstract]) OR (“healthcare acceptance*”[Title/Abstract]) OR (“health technology assess*”[Title/Abstract]) OR (“process model*”[Title/Abstract]) OR (“determinant framework*”[Title/Abstract]) OR (“evaluation framework*”[Title/Abstract]) OR (“consolidated framework for implement*”[Title/Abstract])

9. #7 OR #8

10. #3 AND #6 AND #9

11. “English”[Language]

12. #10 AND #11

13. “2020/01/01”[Date - Publication] : “2024/12/31”[Date - Publication]

14. #12 AND #13

15. (“systematic review”[Publication Type]) OR (“meta-analysis”[Publication Type]) OR (“review”[Publication Type]) OR (“scoping review”[Title/Abstract])

16. #14 NOT #15

17. #14 AND #15

Footnote: additional literature search was conducted with revised publication dates: “2025/01/01”[Date - Publication] : “2026/05/31”[Date - Publication]”.

## Table S1B. Example of PubMed search strategy: CF

1. cystic fibrosis[MeSH Terms]

2. (“cystic fibros*”[Title/Abstract]) OR (“mukovis*”[Title/Abstract]) OR (“mucovis*”[Title/Abstract]) OR (“CF”[Title/Abstract])

Footnote: Steps 3 – 17 are similar to Table S1A.

## Table S1C. Example of PubMed search strategy: COPD

1. (Pulmonary Disease, Chronic Obstructive[MeSH Terms]) OR (pulmonary emphysema[MeSH Terms]) OR (chronic bronchitis[MeSH Terms]) OR (Bronchiectasis[MeSH Terms])

2. (COPD[Title/Abstract]) OR (chronic bronchitis[Title/Abstract]) OR (chronic obstructive airway*[Title/Abstract]) OR (chronic obstructive bronchitis[Title/Abstract]) OR (chronic obstructive bronchopulmonary*[Title/Abstract]) OR (chronic obstructive lung*[Title/Abstract]) OR (chronic obstructive pulmonary*[Title/Abstract]) OR (emphysem*[Title/Abstract]) OR (bronchiectas*[Title/Abstract]) OR (“small airway disease”[Title/Abstract])

Footnote: Steps 3 – 17 are similar to Table S1A.

## Table S1D. Example of PubMed search strategy: COVID-19

1. (coronavirus[MeSH Terms]) OR (Betacoronavirus[MeSH Terms]) OR (coronaviridae infections[MeSH Terms]) OR (SARS-CoV-2[MeSH Terms]) OR (COVID-19[MeSH Terms]) OR (Post-Acute COVID-19 Syndrome[MeSH Terms])

2. (“COVID19*”[Title/Abstract]) OR (“COVID-19”[Title/Abstract]) OR (“2019 ncov*”[Title/Abstract]) OR (“SARS CoV*”[Title/Abstract]) OR (“corona virus*”[Title/Abstract]) OR (“COVID 2019*”[Title/Abstract]) OR (“post covid*”[Title/Abstract]) OR (“long covid*”[Title/Abstract])

Footnote: Steps 3 – 17 are similar to Table S1A.

**Table S2. PRISMA Checklist**

| **Section and Topic** | **Item #** | **Checklist item** | **Location where item is reported** |
| --- | --- | --- | --- |
| **TITLE** | | |  |
| Title | 1 | Identify the report as a systematic review. | Title |
| **ABSTRACT** | | |  |
| Abstract | 2 | See the PRISMA 2020 for Abstracts checklist. | Abstract |
| **INTRODUCTION** | | |  |
| Rationale | 3 | Describe the rationale for the review in the context of existing knowledge. | Introduction, paragraphs 1-7 |
| Objectives | 4 | Provide an explicit statement of the objective(s) or question(s) the review addresses. | Introduction, paragraph 7 |
| **METHODS** | | |  |
| Eligibility criteria | 5 | Specify the inclusion and exclusion criteria for the review and how studies were grouped for the syntheses. | Study Design and Methods: Subsection “Search for review publications and eligibility criteria” and “Two-step review appraisal process and rationale“ |
| Information sources | 6 | Specify all databases, registers, websites, organisations, reference lists and other sources searched or consulted to identify studies. Specify the date when each source was last searched or consulted. | Study Design and Methods: Subsection “Search for review publications and eligibility criteria” |
| Search strategy | 7 | Present the full search strategies for all databases, registers and websites, including any filters and limits used. | Table S1 |
| Selection process | 8 | Specify the methods used to decide whether a study met the inclusion criteria of the review, including how many reviewers screened each record and each report retrieved, whether they worked independently, and if applicable, details of automation tools used in the process. | Study Design and Methods: Subsection “Two-step review appraisal process and rationale“ |
| Data collection process | 9 | Specify the methods used to collect data from reports, including how many reviewers collected data from each report, whether they worked independently, any processes for obtaining or confirming data from study investigators, and if applicable, details of automation tools used in the process. | Study Design and Methods: Subsection “Two-step review appraisal process and rationale“ |
| Data items | 10a | List and define all outcomes for which data were sought. Specify whether all results that were compatible with each outcome domain in each study were sought (e.g. for all measures, time points, analyses), and if not, the methods used to decide which results to collect. | Study Design and Methods: Subsection “Search for review publications and eligibility criteria” and “Two-step review appraisal process and rationale“ |
|  | 10b | List and define all other variables for which data were sought (e.g. participant and intervention characteristics, funding sources). Describe any assumptions made about any missing or unclear information. | Study Design and Methods: Subsection “Framework mapping: Consolidated Framework for Implementation Research (CFIR)”, “Framework mapping: Chaudoir domains” and “Network construction“ |
| Study risk of bias assessment | 11 | Specify the methods used to assess risk of bias in the included studies, including details of the tool(s) used, how many reviewers assessed each study and whether they worked independently, and if applicable, details of automation tools used in the process. | Study Design and Methods: Subsection “Two-step review appraisal process and rationale“ |
| Effect measures | 12 | Specify for each outcome the effect measure(s) (e.g. risk ratio, mean difference) used in the synthesis or presentation of results. | N/A |
| Synthesis methods | 13a | Describe the processes used to decide which studies were eligible for each synthesis (e.g. tabulating the study intervention characteristics and comparing against the planned groups for each synthesis (item #5)). | Study Design and Methods: Subsection “Two-step review appraisal process and rationale“ |
|  | 13b | Describe any methods required to prepare the data for presentation or synthesis, such as handling of missing summary statistics, or data conversions. | Study Design and Methods: Subsection “Two-step review appraisal process and rationale“,“Framework mapping: Consolidated Framework for Implementation Research (CFIR)”, “Framework mapping: Chaudoir domains” and “Network construction“ |
|  | 13c | Describe any methods used to tabulate or visually display results of individual studies and syntheses. | Study Design and Methods: Subsection “Two-step review appraisal process and rationale“,“Framework mapping: Consolidated Framework for Implementation Research (CFIR)”, “Framework mapping: Chaudoir domains” and “Network construction“ |
|  | 13d | Describe any methods used to synthesize results and provide a rationale for the choice(s). If meta-analysis was performed, describe the model(s), method(s) to identify the presence and extent of statistical heterogeneity, and software package(s) used. | Study Design and Methods: Subsection “Two-step review appraisal process and rationale“,“Framework mapping: Consolidated Framework for Implementation Research (CFIR)”, “Framework mapping: Chaudoir domains” and “Network construction“ |
|  | 13e | Describe any methods used to explore possible causes of heterogeneity among study results (e.g. subgroup analysis, meta-regression). | N/A |
|  | 13f | Describe any sensitivity analyses conducted to assess robustness of the synthesized results. | N/A |
| Reporting bias assessment | 14 | Describe any methods used to assess risk of bias due to missing results in a synthesis (arising from reporting biases). | Study Design and Methods: Subsection “Two-step review appraisal process and rationale“ |
| Certainty assessment | 15 | Describe any methods used to assess certainty (or confidence) in the body of evidence for an outcome. | N/A |
| **RESULTS** | | |  |
| Study selection | 16a | Describe the results of the search and selection process, from the number of records identified in the search to the number of studies included in the review, ideally using a flow diagram. | Results: Subsection “Screening of review publications“, “Screening: original publications”, “Included original publications by disease”, “Technology coverage by disease”, and “Network findings” |
|  | 16b | Cite studies that might appear to meet the inclusion criteria, but which were excluded, and explain why they were excluded. | Results: last paragraph in Subsection “Screening: original publications” |
| Study characteristics | 17 | Cite each included study and present its characteristics. | Table 1 |
| Risk of bias in studies | 18 | Present assessments of risk of bias for each included study. | Figure 2 |
| Results of individual studies | 19 | For all outcomes, present, for each study: (a) summary statistics for each group (where appropriate) and (b) an effect estimate and its precision (e.g. confidence/credible interval), ideally using structured tables or plots. | Figures 3-6 |
| Results of syntheses | 20a | For each synthesis, briefly summarise the characteristics and risk of bias among contributing studies. | Results: Subsection “Screening of review publications“, “Screening: original publications”, “Included original publications by disease”, “Technology coverage by disease”, and “Network findings”; Table 1 |
|  | 20b | Present results of all statistical syntheses conducted. If meta-analysis was done, present for each the summary estimate and its precision (e.g. confidence/credible interval) and measures of statistical heterogeneity. If comparing groups, describe the direction of the effect. | Figures 3-6 |
|  | 20c | Present results of all investigations of possible causes of heterogeneity among study results. | N/A |
|  | 20d | Present results of all sensitivity analyses conducted to assess the robustness of the synthesized results. | N/A |
| Reporting biases | 21 | Present assessments of risk of bias due to missing results (arising from reporting biases) for each synthesis assessed. | N/A |
| Certainty of evidence | 22 | Present assessments of certainty (or confidence) in the body of evidence for each outcome assessed. | N/A |
| **DISCUSSION** | | |  |
| Discussion | 23a | Provide a general interpretation of the results in the context of other evidence. | Discussion, paragraphs 1-8 |
|  | 23b | Discuss any limitations of the evidence included in the review. | Discussion, paragraph 9-10 |
|  | 23c | Discuss any limitations of the review processes used. | Discussion, paragraph 10 |
|  | 23d | Discuss implications of the results for practice, policy, and future research. | Discussion, paragraph 11 |
| **OTHER INFORMATION** | | |  |
| Registration and protocol | 24a | Provide registration information for the review, including register name and registration number, or state that the review was not registered. | CRD42025107094 |
|  | 24b | Indicate where the review protocol can be accessed, or state that a protocol was not prepared. | PROSPERO |
|  | 24c | Describe and explain any amendments to information provided at registration or in the protocol. | One amendment submitted following an extension of a screening timeline |
| Support | 25 | Describe sources of financial or non-financial support for the review, and the role of the funders or sponsors in the review. | Section “Funding” |
| Competing interests | 26 | Declare any competing interests of review authors. | Section “Conflict of Interest” |
| Availability of data, code and other materials | 27 | Report which of the following are publicly available and where they can be found: template data collection forms; data extracted from included studies; data used for all analyses; analytic code; any other materials used in the review. | N/A |
